# Estimating the Prevalence of Foodborne Pathogen *Campylobacter jejuni* in Chicken and Its Control via Sorghum Extracts

**DOI:** 10.3390/pathogens12070958

**Published:** 2023-07-20

**Authors:** Gamal M. Hamad, Mariam Gerges, Taha Mehany, Saleh M. Hussein, Michael Eskander, Rasha G. Tawfik, Yasser El-Halmouch, Alaa M. Mansour, Elsayed E. Hafez, Tuba Esatbeyoglu, Eman M. Elghazaly

**Affiliations:** 1 Food Technology Department, Arid Lands Cultivation Research Institute (ALCRI), City of Scientific Research and Technological Applications (SRTA-City), New Borg El-Arab 21934, Egypt; 2 Department of Chemistry, Faculty of Science, Alexandria University, Alexandria 22758, Egypt; 3 Department of Food Science and Technology, Faculty of Agriculture, Al-Azhar University, Assiut 71524, Egypt; 4 Department of Food Hygiene and Control, Faculty of Veterinary Medicine, Alexandria University, Alexandria 22758, Egypt; 5 Department of Microbiology, Faculty of Veterinary Medicine, Alexandria University, Alexandria 22758, Egypt; 6 Department of Botany and Microbiology, Faculty of Science, Kafrelsheikh University, Kafr Elsheikh 33516, Egypt; 7 Department of Animal Hygiene and Zoonoses, Faculty of Veterinary Medicine, Alexandria University, Alexandria 22758, Egypt; 8 Department of Plant Protection and Biomolecular Diagnosis, Arid Lands Cultivation Research Institute (ALCRI), City of Scientific Research and Technological Applications (SRTA-City), New Borg El-Arab 21934, Egypt; 9 Department of Food Development and Food Quality, Institute of Food Science and Human Nutrition, Gottfried Wilhelm Leibniz University Hannover, Am Kleinen Felde 30, 30167 Hannover, Germany; 10 Department of Microbiology, Faculty of Veterinary Medicine, Matrouh University, Mersa Matruh 51511, Egypt

**Keywords:** *Campylobacter jejuni*, foodborne, natural antioxidant, antibacterial activity, natural preservatives, polyphenols, sorghum extract, chicken fillet

## Abstract

*Campylobacter jejuni* is a Gram-negative bacterium which is considered as the most reported cause of foodborne infection, especially for poultry species. The object of this work is to evaluate the occurrence of *C. jejuni* in chicken meat as well its control via three types of sorghum extracts (white sorghum (WS), yellow sorghum (YS), and red sorghum (RS)); antibacterial activity, antioxidant power, and cytotoxicity of sorghum extracts were also assessed. It was found that *C. jejuni* is very abundant in chicken meat, especially breast and thigh. WS extract showed more effectiveness than both yellow and red ones. Lyophilized WS extract offered high total phenolic compounds (TPCs) and total flavonoid compounds (TFCs) of 64.2 ± 0.8 mg gallic acid equivalent (GAE/g) and 33.9 ± 0.4 mg catechol equivalent (CE)/g, respectively. Concerning the antibacterial and antioxidant activities, WS showed high and significant antibacterial activity (*p* < 0.001); hence, WS displayed a minimum inhibitory concentration (MIC) of 6.25%, and revealed an inhibition zone of 7.8 ± 0.3 mm; it also showed an IC_50_ at a concentration of 34.6 μg/mL. In our study, different samples of chicken fillet were collected and inoculated with pathogenic *C. jejuni* and stored at 4 °C. Inoculated samples were treated with lyophilized WS extract at (2%, 4%, and 6%), the 2% treatment showed a full reduction in *C. jejuni* on the 10th day, the 4% treatment showed a full reduction in *C. jejuni* on the 8th day, while the 6% treatment showed a full reduction in *C. jejuni* on the 6th day. Additionally, 2%, 4%, and 6% WS extracts were applied on un-inoculated grilled chicken fillet, which enhanced its sensory attributes. In sum, WS extract is a promising natural preservative for chicken meat with accepted sensory evaluation results thanks to its high antibacterial and antioxidant potentials.

## 1. Introduction

*Campylobacter jejuni* is a Gram-negative, microaerophilic bacterium that is regarded as a worldwide leading cause of foodborne illness [1]. It is responsible for most bacterial infections associated with poultry consumption, causing symptoms ranging from mild diarrhea to severe abdominal cramping and fever [2,3]. *Campylobacter* infections were found to cause several diarrheal diseases from 2 to 7 times as regularly as infections with *Escherichia coli*, *Salmonella* species, or *Shigella* species [4]. The bacterium is expected to be responsible for over 400 million cases of gastroenteritis annually; most of these cases are in developing countries [5].

Poultry is considered a primary host for *C. jejuni*, with up to 90% of chicken and turkey flocks being colonized with the bacterium. The contamination of poultry meat during processing is a considerable concern, as it can cause transmission of various bacteria to humans [6,7]. The occurrence of *C. jejuni* in poultry is influenced by a range of diverse factors, including the hygiene of the rearing environment, the use of antibiotics, and the presence of other microorganisms [8,9,10]. The high occurrence of *Campylobacter* species including *C. jejuni* that mainly invade poultry species has been reported to be due to its ability to attack birds’ intestinal tracts’ epithelium and multiply inside it, owing to their warm-blooded body nature [11,12]. Chicken ceca of age between 14 and 21 days are colonized by *C. jejuni* reaching 1 × 10^9^ CFU/g [13]. The ability of the bacterium to form biofilms also contributes to its persistence in the poultry gut, as these structures provide a protective environment that can resist antimicrobial treatments [14].

Recently, consumers’ demands regarding healthy and sustainable food have increased and are currently more critical than before owing to the severe outcomes of epidemics, climate change, and conflicts [15]. Additionally, there is a serious need for efficient interventions to minimize the occurrence of pathogenic bacteria in poultry species, providing that *C. jejuni* contributes most of the pathogenic contamination in poultry species that causes dangerous public health consequences [16]. Lately, it was found that sorghum, which is a cereal crop which belongs to the family Graminae, has been found to possess anti-bacterial properties against a range of pathogenic bacterial species. Sorghum plants have been identified as potential sources of natural antimicrobial compounds due to the existence of high levels of phytochemicals, such as tannins and flavonoids [17]. Furthermore, sorghum extracts have been shown to have a prebiotic effect, promoting the growth of beneficial gut bacteria while inhibiting the growth of harmful pathogens [18].

In this paper, the extracts of three types of sorghum were employed as antibacterial agents against *C. jejuni*, as it has been reported recently that sorghum extract can be used as a natural alternative to antibiotics in poultry feed to control *C. jejuni* colonization in the intestinal tract of broiler chickens, which could ultimately minimize the risk of human infection [19]. Therefore, the aim of our study is the experimental application of sorghum extract on chicken fillet inoculated experimentally with *C. jejuni*, and assessment of its antibacterial activity, antioxidant activity, minimum inhibitory concentration (MIC), total phenolic compounds (TPCs), total flavonoid compounds (TFCs), and the cytotoxicity.

## 2. Materials and Methods

### 2.1. Growth Conditions of Bacterial Strains

The *C. jejuni* EMCC 1835 reference strain was brought from Microbial Resource Center (MIRCEN), Ain Shams University, Cairo, Egypt. The cell counts were accustomed to 10^6^ CFU/mL as the infective dose is >10^5^ CFU/g [20]. The strains were stored at a temperature of −80 °C in Brain Heart Broth (Merck, 1.10493.0500, Darmstadt, Germany), including both glycerol and lysed horse blood (LHB) (Oxoid, SR048C, Hampshire, UK), then they were sub-cultured at a temperature of 42 °C on Columbia agar base (CAB) (Oxoid, CM0331, Hampshire, UK) under certain conditions as follow: 5% O_2_, 85% N_2_, and 10% CO_2_.

### 2.2. Collection of Chicken Samples and Detection of C. jejuni

A hundred chicken breast, thigh, liver, and gizzard samples were grouped and gathered from various local markets located in Alexandria Governorate, Egypt, in 2022. During the collection process, the chicken meat that is sold in pieces was randomly collected from the local retail stores at refrigerated temperature and packaged in bags made of polyethylene. Immediately after collection, samples were transferred in a box containing ice to the laboratory to be examined bacteriologically. *C. jejuni* was isolated and applied according to the method illustrated by El-Khawas et al. [21].

### 2.3. Sorghum Materials and Extraction

**Figure 1:**
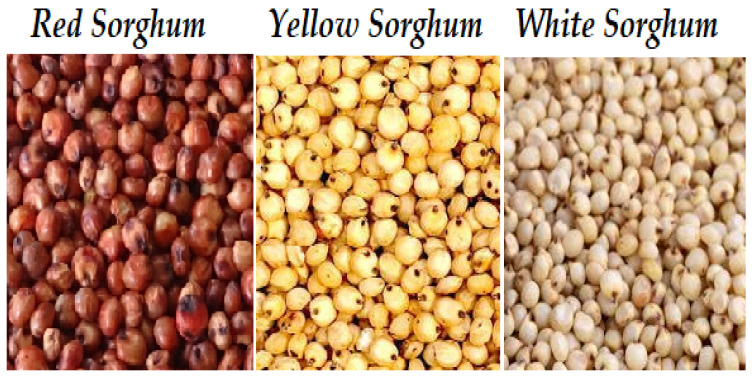
Pictures of the investigated sorghum seeds in this study: red, yellow, and white sorghum seeds (*Sorghum bicolor* L.).

Three local varieties of sorghum—red, white, and yellow seeds (see Figure 1)—were obtained and identified by the Shandawil Sohag Research Center (Sohag Governorate, Egypt) as follows: Shandaweel 1 (white sorghum), Giza 54 (yellow sorghum), Alsabeinaa (red sorghum). The collected grains were washed and dried, and 10 g were then extracted in an ethanolic solution (70% ethanol: deionized water *v*/*v*) of up to 100 mL with random shaking at 100 rpm. After incubation for 2 days, the attained extracts were centrifuged for 30 min at 2.147× *g*, then filtered by using normal filter papers. Finally, extract lyophilization took place at −50 °C (Telstar Model 50, Barcelona, Spain) and the resultant extracts were diluted in distilled H_2_O with specific recognized concentrations in mg/mL [22,23].

### 2.4. Antibacterial Activity

#### 2.4.1. Antibacterial Potential of Sorghum Extract

The aptitude of sorghum extracts as a microbial agent contrary to *C. jejuni* EMCC 1835 reference strain (prepared in MIRCEN) was measured following the procedure of Hamad et al. [22] and Klančnik et al. [24]. The overnight *C. jejuni* cultures were enriched on Mueller hinton media (MHM) (Oxoid, UK) at 42 °C/48 h and then spread on MHM plates. The inhibition zone was then recorded (mm), observing the anti-*C. jejuni* power of the three types of sorghum extracts. Additionally, a comparative study was carried out between the results of the inhibition zone and those of 3 antibiotic disks which are Erythromycin (ERY), Gentamicin (GEN), and Amoxicillin (AMX) [25].

#### 2.4.2. Assessment MICs of Sorghum Extract against *C. jejuni*

Sorghum extracts of minimum inhibitory concentrations against *C. jejuni* EMCC 1835 were evaluated using agar disk diffusion assay [22,25] with the following descending concentrations of white sorghum extract: 100, 50, 25, 12.5, 6.25, 3.12 µL. *C. jejuni* spread on MHM plates of grown Mueller Hinton Medium (MHM) plates and adjusted to a density of 10^6^ CFU/mL [26] and the plates were kept at a temperature of 4 °C for 30 min and then incubated at 42 °C/24 h. A clear and sharp inhibition zone was noted in mm, taking in consideration the anti-*C. jejuni* potential of the different sorghum extracts.

### 2.5. Phytochemical Analysis of White Sorghum Extract

#### 2.5.1. Total Phenolic Compounds (TPCs) of White Sorghum Extract

The total phenol compounds (TPCs) of sorghum extracts was evaluated by using the test method described by Hamad et al. [23]. Briefly, approximately 0.1 mL reconstituted extract was incorporated into a 100 µL Folin–Ciocalteu substance, the mixture then stood for fifteen min, and then we added 2 mL sodium carbonate (2%). After that, the mixture was left at ambient temperature for half hour, and gallic acid as a calibration was used in the evaluation of TPCs by using a spectrophotometer (T80, PG Instrument, Lutterworth, UK) at 760 nm. TPC is expressed as mg of gallic acid equivalents to each gram per sample.

#### 2.5.2. Total Flavonoid Compounds (TFCs) of White Sorghum Extract

TFCs of sorghum extracts was assessed following the method illustrated by Hamad et al. [27]. Four milliliters of water was mixed with 1 mL of the examined sample in a volumetric flask. After that, 0.15 mL of aluminum chloride (10% AlCl_3_) and 0.75 mL of sodium nitrite (5% NaNO_2_) were added. Finally, and after 5 min, 500 µL of 1 M from NaOH was added. The TFCs was measured spectrophotometrically at 510 nm.

#### 2.5.3. Diphenyl-1-Picrylhydrazyl (DPPH) Radical Scavenging Assay

The ability of sorghum extracts to scavenge DPPH free radicals was examined [27,28] by applying some modifications. A stock solution of each extract in methanol to 1 mg/mL was prepared. Serialized dilutions of plant extracts were carried out by mixing about 1 mL of every dilution with 1 mL DPPH and then measured at 517 nm spectrophotometrically. The results were expressed as IC_50_ (the concentration of the extract that can suppress the 50% DPPH). Then we calculated inhibition % through this equation:

*DPPH inhibition* % = [(*A of control* − *A of the sample*)/*A of control*] × 100 (1)

where: *A*: Absorbance.

### 2.6. Safety and Cytotoxicity Assay of White Sorghum Extract

White sorghum extract was assessed for its impact on the peripheral blood mononuclear cell’s viability (PBMCs). Tested wells (150 µL PBMCs), control wells (150 µL PBMCs), and blank wells (150 µL PBS) were used. Several different concentrations of white sorghum extract were inoculated for test wells and incubated for one day, according to the approach of Popiołkiewicz et al. [29]. Using the spectrophotometer, the absorbance was observed at 540 nm; white sorghum extract inhibition % was calculated from the following equation. IC_50_ values were calculated through this portal www.aatbio.com/tools/ic50-calculator (accessed on 22 February 2023).

*White sorghum extract inhibition* % = 100 − (*optical density* (*O.D*) *Control* − *O.D Treatment*/*O.D Control*) (2)

### 2.7. Experimental Application and Evaluation of the Antimicrobial Power of White Sorghum Extract against C. jejuni Experimentally Inoculated into Chicken Fillet

#### 2.7.1. Microbes

*C. jejuni* EMCC 1835 was gained from MIRCEN. Bacterial strain was set, and its bacterial density was adjusted at a value of 1 × 10^7^ CFU/mL [26,30].

#### 2.7.2. Refrigerated Storage Study of Chicken Breast Fillets

Raw boneless chicken breast fillets were sliced with a sterile knife into 5 cm × 5 cm pieces. Before doing the experiment, chicken samples were sterilized according to Hamad et al. [22] and Morsy et al. [31]. Chicken samples were grouped into several treatments (6 treatments): T1, chicken samples without applying any handlings (negative control (1)); T2, chicken samples supplemented with white sorghum extract (2%) (negative control (2)); T3, chicken samples with 1 × 10^7^ CFU/mL *C. jejuni* (positive control); T4, chicken samples with *C. jejuni* and white sorghum extract (2%); T5, chicken samples with *C. jejuni* and white sorghum extract (4%); T6, chicken samples with *C. jejuni* and white sorghum extract (6%).

Samples were kept for a time of 15 min at room temperature, and further were cooled at 4 °C and observed for *C. jejuni* occurrence every two days bacteriologically until complete reduction of the bacterial count. Chicken samples were assessed bacteriologically at various storage periods (i.e., 0, 2, 4, 6, 8, and 10 days) for determination of *C. jejuni* amount in the products [32].

#### 2.7.3. Sensory Evaluation of the Acceptability of Chicken Fillet Fortified with White Sorghum Extract

To determine the acceptability of white sorghum extract as a food additive, organoleptic attributes were evaluated in chicken samples enriched with the sorghum extracts. The experiment was carried out on four groups, the first group is chicken fillet without any treatments (control); the other three groups are chicken samples with different concentrations of white sorghum extract of 2%, 4%, and 6%. Ten experienced panelists analyzed the samples. Panelists checked the degree of acceptability of chicken for the sensorial scores: texture, appearance, taste, odor, color, and overall acceptability, with a scale ranging from 1 to 10 (10 points/each item), where 10 is more accepted [22,33].

### 2.8. Statistical Analysis

R software, version 4.2.0 (Lucent Technologies, New Providence, NJ, United States), was employed for data analyses. One-way analysis of variance (ANOVA) of the means of three reads (mean ± SE) using Tukey’s test at *p* < 0.01 or *p* < 0.05 and regression analysis was used for continuous independent variables.

## 3. Results and Discussion

### 3.1. Occurrence of C. jejuni in Chicken Meat

Chicken meat makes up a high percentage of our diet, which surely leads to human infection. Unfortunately, the processing step is the main reason various poisoning bacteria contaminate chicken food products [22]. The occurrence of *C. jejuni* in chicken fillets can vary between countries and regions and has been the subject of numerous studies. *C. jejuni* affects human health negatively as it causes mild to severe symptoms such as diarrhea, fever, nausea, and vomiting. Symptoms typically develop 2–5 days after exposure to the bacteria and can last for up to 10 days [34].

In our study, the collected chicken meat samples showed a high presence of *C. jejuni* especially in thigh and breast meat with percentages of 88% and 80%, respectively, as illustrated in Table 1. These findings are in line with results shown by Walker et al. [35] who isolated *E. coli* and *C. jejuni* from chicken, pork offal, lamb, and beef collected from retail food outlets and showed a very high incidence of *Campylobacter* in chicken meat.

The chicken meat samples in this study were collected from suppliers with poor hygiene levels and low sanitation levels for chicken cutting tools to observe the prevalence of *C. jejuni*. In addition, the high incidence of *C. jejuni* found in chicken samples may be caused by contamination from microbes anywhere along the supply chain. Therefore, this highlights the importance of implementing proper hygiene and sanitation practices to prevent contamination and the spread of foodborne illness. Additionally, consumers should be aware of the risks associated with improperly handled and cooked chicken meat and should follow proper food safety guidelines to minimize the risk of foodborne illness.

### 3.2. Antibacterial Activity of Lyophilized Sorghum Extracts

**Figure 2:**
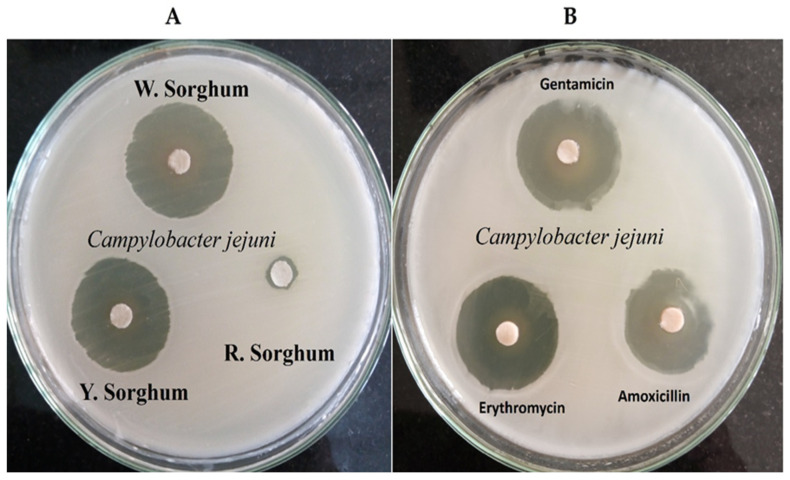
Antibacterial activity of red (R. Sorghum), white (W. Sorghum), and yellow sorghum (Y. Sorghum) extracts against *C. jejuni* using agar disk diffusion assay vs. Erythromycin, Gentamicin, and Amoxicillin antibiotics. Inhibition zones are indicated in mm. (**A**) Antibacterial impact of three sorghum extracts; (**B**) Antibacterial power of 3 antibiotics compared to sorghum extracts.

Three types of sorghum extracts were prepared and the antibacterial effect on *C. jejuni* was evaluated using agar disk diffusion assay as well as three types of antibiotics: Gentamicin, Erythromycin, and Amoxicillin. Then, results were obtained; they are shown in Figure 2 and tabulated in Table 2. 

Results showed that red sorghum extract has a negative effect on *C. jejuni*, while the other sorghum extracts showed significant effects, especially white sorghum which exhibited an inhibitory zone of 39.1 ± 0.2 mm, even higher than the three antibiotic types (*p* < 0.001). White and yellow sorghum showed extremely high effects compared with Piskernik et al. [36], who used rosemary extract. A recent study by Chen et al. [37] reported that polyphenol extract of sweet sorghum displayed antibacterial activity against *Staphylococcus aureus*, *Escherichia coli*, *Listeria* spp., and *Salmonella* spp. Another report by Garzón et al. [38] confirmed that a higher antimicrobial potential of sorghum spent grain could be an important natural source of bioactive peptides with antimicrobial activity against *Bacillus cereus* growth.

### 3.3. Minimal Inhibitory Concentration (MIC) of Sorghum Plant Extract

**Figure 3:**
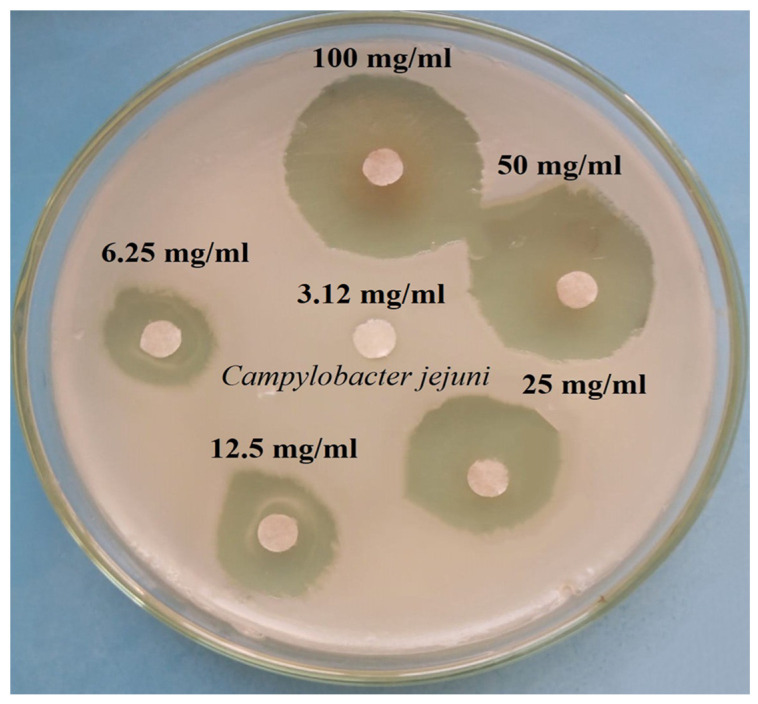
Minimum inhibitory concentrations (MICs) of the white sorghum extracts against *C. jejuni* (mm).

In our study, the MIC of lyophilized white sorghum extract against *C. jejuni* in vitro and the antimicrobial potential at several ratios of white sorghum extracts were examined; the results are shown in Figure 3 and tabulated in Table 3. The results confirmed that the MIC value of white sorghum extract was 6.25% with an inhibition zone of 7.8 ± 0.3 mm. Additionally, the concentration of 3.12% showed negative results and the anti-*C. jejuni* bacterial activity increased gradually on increasing the extract concentration percentage (*p* < 0.001).

### 3.4. Total Phenolic Compounds (TPCs) and Total Flavonoid Compounds (TFCs) of Lyophilized Sorghum Extract

Phenolic compounds and flavonoids are important antioxidant and antibacterial agents in plants that play a significant role in preventing many diseases such as cancer and promote human health and immunology [39].

According to the results in Table 4, TPC and TFC of white sorghum extract were 64.2 ± 0.8 mg GAE/g and 33.9 ± 0.4 mg CE/g, respectively, so it has the highest antioxidant activity. It showed significant results, followed by yellow sorghum extract then red sorghum extract. White sorghum extract showed the best results in treating *C. jejuni* in chicken fillets.

TPC and TFC values may differ according to temperature, seasonal exchange, pH, polarity of used solvents in extraction process, light incidence, water nutrient composition, and salinity [22,40,41].

### 3.5. Antioxidant Potential and DPPH Radical Scavenging Ability

The antioxidant power can be estimated accurately via assessment of the DPPH radical scavenging ability. The antioxidant capacity of lyophilized sorghum extract was estimated according to the DPPH radical scavenging capacity and compared with a standard which is ascorbic acid. Results are presented in Table 5; it was observed that the IC_50_ values of the lyophilized extracts were 34.62 μg/mL for white sorghum, 51.5 μg/mL for yellow sorghum, and 65.8 μg/mL for red sorghum, while the IC_50_ value of ascorbic acid was 20.1 μg/mL. The highest free radical scavenging capacity of the extracts was 99.2% at a ratio of 100 μg/mL for white sorghum, 97.6% at a concentration of 100 μg/mL for yellow sorghum, and 89.2% at a concentration of 100 μg/mL for red sorghum. These findings contradict the findings of Pontieri et al. [42], who found that red sorghum extract has higher antioxidant activity than white sorghum.

Some sorghum species contain several types of phytochemicals that have the ability to neutralize free radicals. It has been confirmed that the high antioxidant activity of sorghum extract gives it a potential health effect as protection against cardiovascular disease, obesity, dyslipidemia, oxidative stress, and diabetes [42,43], as well as antimicrobial, anti-inflammatory, and anticancer activity [42,44].

### 3.6. Safety Assay and Cytotoxicity of White Sorghum Extract

The PBMC cytotoxicity method involves using a number of cells obtained from various individuals to assess the in vitro cytotoxicity of potential drugs in a high-throughput manner. Moreover, this assay can offer valuable insights into the response of immune cells from different donors to the compounds being developed. By utilizing this approach, researchers can obtain a more comprehensive understanding of the efficacy and safety of potential drugs [22]. That is why both the safety and the cytotoxicity of lyophilized white sorghum extract were assessed due to the high concern regarding evaluation of the safety of new antimicrobials applied on food. The estimated cytotoxic effects of white sorghum extract on peripheral blood mononuclear cell (PBMC) viability and IC_50_ are presented in Table 6 and exhibited a positive correlation with the concentration of sorghum extract. The concentration ranged from 19.5 µg/mL to 10,000 µg/mL. It was proven that the lyophilized extract is toxic to PBMCs at several concentrations as the minimum concentration showed 16% inhibition and 84% viability, while the maximum concentration showed 100% inhibition and zero viability. Furthermore, white sorghum showed a high IC_50_ at 482.4 µg/mL, which allowed its usage as a safe and promising food additive in chicken meat.

### 3.7. Preparation of Chicken Fillets and Their Acceptability after the Fortification with Lyophilized White Sorghum Extract

Gaining insight into how consumers perceive the safety and quality of chicken meat can be a valuable resource for public health educators, as it is the most widely consumed meat [45]. To ensure the safety of chicken meat upon applying the white sorghum extract, experimentally inoculated chicken fillets with *C. jejuni* were prepared and treated with different concentrations of white sorghum extract to determine the antibacterial effect. In Table 7, the results are presented; they reveal that white sorghum extract has an anti-*C. jejuni* effect on chicken fillet meat stored at 4 °C. The 6% treatment showed a high chicken fillet treatment and a complete reduction of *C. jejuni* on the 6th day, while the 2% treatment showed a low chicken fillet treatment and a complete reduction of *C. jejuni* on the 10th day.

**Figure 4:**
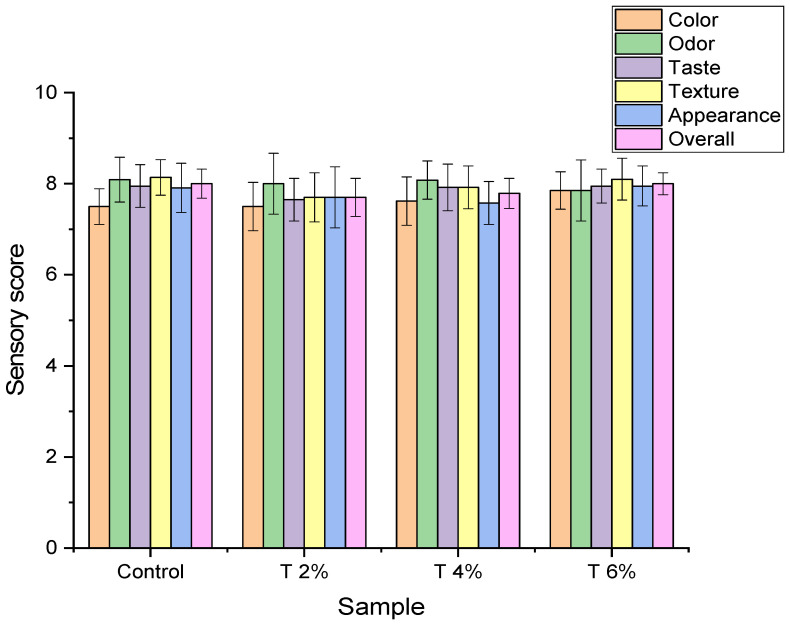
Grilled un-inoculated chicken fillet’s acceptability supplemented with white sorghum extract based on organoleptic characteristics. Control: chicken fillet without any treatment; T 2%: chicken with white sorghum extract 2%; T 4%: chicken with white sorghum extract 4%; T 6%: chicken with white sorghum extract 6%.

In addition to the above study, sensory attributions of grilled un-inoculated chicken fillet were assessed upon applying white sorghum extract. Results are tabulated in Figure 4 and showed that white sorghum of 2%, 4%, and 6% improved taste, color, appearance, odor, and texture of the grilled chicken fillet. In addition to being a good preservative for chicken fillet due to its high antibacterial activity as shown before, it was reported that sorghum could increase the nutritional value of food due to its enrichment with proteins, vitamins including riboflavin, vitamin B6, thiamin, and several minerals including sodium, potassium, zinc, and iron [46]. It is also reported that sorghum flour has a water-holding capacity and has the ability to lower meat fat content so it could improve the texture of chicken fillet meat and increase its juiciness [47].

## 4. Conclusions

In conclusion, *C. jejuni* is very abundant in chicken meat, especially breast and thigh. WS extract showed more effectiveness than both yellow and red ones. WS extract showed a high antibacterial effect against *C. jejuni* due to its large TPC and TFC content, in addition to its high antioxidant activity that could scavenge free radicals. Inoculated samples were treated with lyophilized WS extract at several ratios; the 2% treatment showed a full reduction of *C. jejuni* on the 10th day, the 4% treatment showed a full reduction of *C. jejuni* on the 8th day, and the 6% treatment showed a full reduction of *C. jejuni* on the 6th day. Furthermore, WS extract showed a high acceptance upon application to chicken fillet as it does not affect negatively its sensory attributes. The current results confirmed that WS can be used as a promising food preservative because it is safe and is not toxic for humans; moreover, it increases the nutritional value of food and improves both the texture and the juiciness of meat.

## Figures and Tables

**Table 1:** Incidence of *C. jejuni* in chicken collected from different local markets (*n* = 100).

Samples		Positive Samples	
Types of Chicken Fillet	No. of Samples	No.	%
Breast	25	20	80
Thigh	25	22	88
Liver	25	15	60
Gizzard	25	18	72
Total	100	75	75

**Table 2:** Antibacterial activity of lyophilized sorghum extracts against *C. jejuni* using agar disk diffusion assay.

Sorghum Extracts/Antibiotics	Concentration	Inhibition Zone Diameter (mm)
White sorghum extracts	100 mg/mL	39.1 ± 0.2 ^a^
Yellow sorghum extracts	100 mg/mL	30.1 ± 0.2 ^c^
Red sorghum extracts	100 mg/mL	ND
Gentamicin (GEN)	30 mg/mL	35.3 ± 0.1 ^b^
Erythromycin (ERY)	100 mg/mL	29.1 ± 0.1 ^cd^
Amoxicillin (AMX)	30 mg/mL	26.4 ± 0.1 ^e^

ND, not detected; Data represented are the means of triplicates ± standard error of means; ^a, b, c, d, e^ Mean values with different superscript letters in the same column are significantly different (*p* < 0.001); Depending on the in vitro antibacterial evaluation results, extracts were chosen for further application in chicken fillet against *C. jejuni.*

**Table 3:** Minimum inhibitory concentrations (MICs) of the white sorghum extracts against *C. jejuni* (mm).

**Strains/Extract**	**White Sorghum Extracts (mg/mL)**	
	**Conc. (%)**	**Inhibition zone (mm)**
	100	38.7 ± 0.6 ^a^
	50	24.8 ± 0.8 ^b^
	25	16.1 ± 0.7 ^c^
	12.5	11.8 ± 0.8 ^d^
	6.25	7.8 ± 0.3 ^e^
	3.12	ND

MIC, minimum inhibitory concentration; ND, not detected; Data represented are the means of triplicates ± standard error of means; ^a, b, c, d, e^ Mean values with different superscript letters in the same column are significantly different (*p* < 0.001).

**Table 4:** Total phenolic (mg GAE/g) and flavonoid contents (mg CE/g) of sorghum extracts.

Extracts	Total Phenolic Content (mg GAE/g)	Total Flavonoid Content (mg CE/g)
White sorghum extract	64.2 ± 0.8 ^a^	33.9 ± 0.4 ^a^
Yellow sorghum extract	31.6 ± 1.4 ^b^	21.9 ± 0.4 ^b^
Red sorghum extract	15.5 ± 0.5 ^c^	7.20 ± 0.3 ^c^

Data represented are the means of triplicates ± standard error of means; ^a, b, c^ Mean values with different superscript letters in the same column are significantly different (*p* < 0.001).

**Table 5:** Antioxidant activity and DPPH radical scavenging capacity of the sorghum extracts.

Conc. (μg/mL)	Ascorbic Acid		White Sorghum Extract		Yellow Sorghum Extract		Red Sorghum Extract	
	Inhibition (%)	IC_50_ (μg/mL)	Inhibition (%)	IC_50_ (μg/mL)	Inhibition (%)	IC_50_ (μg/mL)	Inhibition (%)	IC_50_ (μg/mL)
10	33.2 (0.0) ^a^	20.1	25.6 (0.0) ^b^	34.6	11.3 (0.0) ^c^	51.5	5.2 (0.0) ^d^	65.8
20	49.8 (0.0) ^a^		34.6 (0.0) ^b^		19.2 (0.0) ^c^		12.5 (0.0) ^d^	
30	78.4 (0.0) ^a^		43.3 (0.0) ^b^		27.3(0.0) ^c^		18.6(0.0) ^d^	
40	84.5 (0.0) ^a^		51.4 (0.0) ^b^		36.3 (0.0) ^c^		28.2 (0.0) ^d^	
50	90.7 (0.0) ^a^		63.6 (0.0) ^b^		48.5 (0.0) ^c^		35.3 (0.0) ^d^	
60	93.3 (0.0) ^a^		75.2 (0.0) ^b^		56.9 (0.0) ^c^		45.6 (0.0) ^d^	
70	95.3 (0.0) ^a^		92.5 (0.0) ^b^		71.2 (0.0) ^c^		55.5 (0.0) ^d^	
80	97.4 (0.0) ^a^		95.2 (0.0) ^b^		86.4 (0.0) ^c^		69.2 (0.0) ^d^	
90	98.4 (0.0) ^a^		97.3 (0.0) ^ab^		95.7 (0.0) ^c^		83.4 (0.0) ^d^	
100	99.2 (0.0) ^a^		98.8 (0.0) ^ab^		97.6 (0.0) ^c^		89.2 (0.0) ^d^	

Data represented are the means of triplicates ± standard error of means; ^a, b, c, d^ Mean values with different superscript letters in the same row are significantly different (*p* < 0.001).

**Table 6:** The safety and cytotoxicity assessment of white sorghum extract on the viability of PBMC cells.

Concentration (µg/mL)	Inhibition %	Viability %
10,000	100	0
5000	100	0
2500	97	3
1250	88	12
625	69	31
312	53	47
156	43	57
78	32	68
39	28	72
19.5	16	84
IC_50_ = 482.4		

**Table 7:** Antibacterial impact of several ratios from white sorghum extract against *C. jejuni* experimentally inoculated into chicken fillet stored at 4 °C (mean ± SE).

Storage (Days)	Negative Control (1)	Negative Control (2)	Positive Control	Treatment (2%)	Treatment (4%)	Treatment (6%)
0	0.00	0.00	1 × 10^7^	1 × 10^7^ (0.0)	1 × 10^7^ (0.1)	1 × 10^7^ (0.1)
2nd	0.00	0.00	1 × 10^7^	1.81 × 10^6^ (0.0)	6.21 × 10^5^ (0.0)	4.1 × 10^4^ (0.1)
4th	0.00	0.00	1 × 10^7^	7.21 × 10^5^ (0.0)	2.51 × 10^4^ (0.0)	2.1 × 10^2^ (0.0)
6th	0.00	0.00	1 × 10^7^	3.21 × 10^4^ (0.0)	1.4 × 10^2^ (0.0)	0.00 (0.0)
8th	0.00	0.00	1 × 10^7^	1.8 × 10^6^ (0.0)	0.00 (0.0)	0.00 (0.0)
10th	0.00	0.00	1 × 10^7^	0.00 (0.0)	0.00 (0.0)	0.00 (0.0)

*C. jejuni* counts are in (Log10 CFU/g); Data represented are the means of triplicates ± standard error of means; All data have a total significant difference of *p* < 0.001.

## Data Availability

The data presented in this study are available upon request from the corresponding author.
